# Report of incomplete intestinal obstruction in an adult patient with Down Syndrome: A literature review

**DOI:** 10.12669/pjms.40.9.8777

**Published:** 2024-10

**Authors:** Ning Yang

**Affiliations:** Ning Yang, Department of General Surgery, The First Affiliated Hospital, Hebei Medical University, Shijiazhuang 050030, Hebei, China

**Keywords:** Adult, Down syndrome, Intestinal obstruction, Adhesions, Laparoscopic examination

## Abstract

We report a case of incomplete intestinal obstruction in a 29-year-old adult male with Down syndrome in The First Affiliated Hospital, Hebei Medical University from January 2023 to July 2023 the patient had been experiencing intermittent abdominal distension and reduced bowel movements and gas for a period of two years. Physical examination revealed tenderness in the left lower abdomen. Abdominal CT and gastrointestinal imaging showed intestinal and colonic distension, while colonoscopy did not show any abnormalities. During laparoscopic exploration, adhesions were observed between the greater omentum and the left lower abdominal wall, resulting in dilation of the proximal colon. After the adhesions were released, intestinal contents were able to pass through the distal segment, relieving the obstruction. The patient had no history of trauma or surgery. This case highlights a potential association between Down syndrome and congenital abdominal adhesions that can lead to intestinal obstruction. Due to the communication difficulties in patients with Down syndrome, there is a potential for misdiagnosis, emphasizing the importance of awareness. Laparoscopic examination not only aids in the diagnosis but also offers therapeutic benefits in such cases.

## INTRODUCTION

Down syndrome (DS) is a congenital genetic disorder caused by an abnormality in chromosome 21. The incidence of DS increases with maternal age and varies among different populations.^1^ Individuals with DS typically display distinctive facial features, intellectual disabilities, and developmental delays. Compared to the general population, individuals with DS have an increased susceptibility to various medical conditions, including congenital heart disease, obstructive sleep apnea, leukemia, diabetes, and Alzheimer’s disease.^2^,^3^ Gastrointestinal problems, such as reflux, obesity, constipation, and diarrhea, are also commonly observed in adult patients with DS.^4^ In this report, The First Affiliated Hospital, Hebei Medical University present a case of an adult patient with DS who experienced recurrent episodes of incomplete intestinal obstruction.

## CASE PRESENTATION

**Fig.1:**
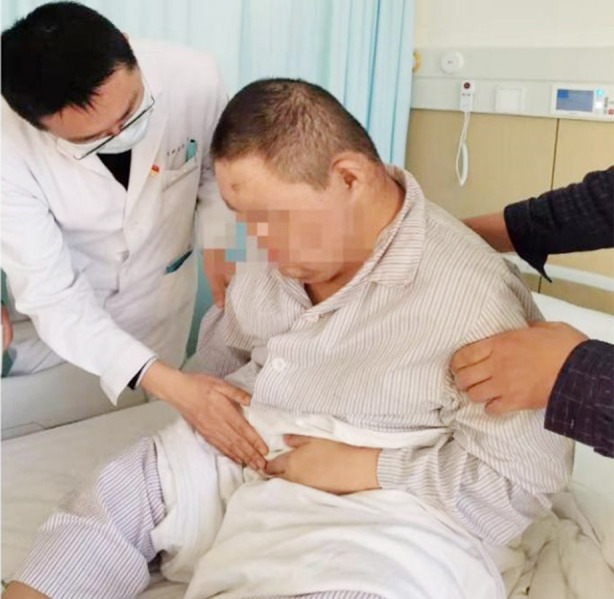
The patient exhibits typical signs of Down syndrome (DS).

The patient is a 29-year-old male with intellectual functioning equivalent to that of a 7-8-year-old child. He has basic literacy skills and is able to communicate in simple terms. He has a preference for greasy foods, tends to overeat, and has a sedentary lifestyle. At the age of one, he was diagnosed with DS at a local hospital, and he exhibits typical physical features associated with DS (Fig.1).

### Ethical Approval:

We have taken permission to use the photograph of the patient. The study was approved by the Institutional Ethics Committee of The First Affiliated Hospital, Hebei Medical University (No.: [2023]S00821; date: July 13, 2023), and written informed consent was obtained from participants.

Two years ago, the patient experienced intermittent abdominal distension without any apparent trigger. The discomfort was predominantly localized to the left lower abdomen and did not radiate to other areas. The duration of the pain varied between five and fifteen minutes, with intervals ranging from several hours to a few days, without a regular pattern. He also experienced accompanying symptoms of nausea and non-projectile vomiting, with the vomitus containing gastric contents. Additionally, he had a reduction in passing gas and bowel movements. The patient was initially diagnosed with “incomplete intestinal obstruction” at a local hospital and showed improvement after conservative treatments such as enemas and fluid replacement. The symptoms recurred one year ago and again six months ago, with similar characteristics, and improved following conservative treatment. However, in the two months leading up to admission, the episodes became more frequent, with an increased duration of 30 to 120 min. and worsened intensity compared to before.

The patient had a history of alternating constipation and diarrhea for five years. He was diagnosed with “Type-2 diabetes” two years ago and was taking metformin extended-release tablets (0.5 g, twice daily). His fasting blood glucose levels were around 8.0 mmol/L, and postprandial blood glucose levels were around 8.6 mmol/L. The patient denied any history of surgery or trauma. There was no family history of DS.

Physical examination revealed an obese patient with a BMI of 31.1 kg/m^2^. He had a dull expression, lethargy, a flexed posture, a round face, small external ears, wide intercanthal distance, a flat nose, thick lips, simian crease, short fingers, and short limbs. The abdomen was slightly distended, without visible peristalsis or visible superficial abdominal veins. Tenderness was present in the left lower abdomen, with a fixed tender point, but no rebound tenderness or muscle guarding. Bowel sounds were active.

### Auxiliary examinations:

Blood routine examination: WBC 7.0×10^9/L, HGB 138 g/L, PLT 291×10^9/L. Biochemical examination: Ca^2+^ 2.23 mmol/L, CL^-^ 91.3 mmol/L, Na^+^ 130.0 mmol/L K^+^ 4.26 mmol/L, TP 64.2 g/L, ALB 40.0 g/L, BS 10.65 mmol/L. Abdominal and pelvic CT scans revealed colonic distension with intraluminal gas.

### Diagnostic and therapeutic process:

**Fig.2:**
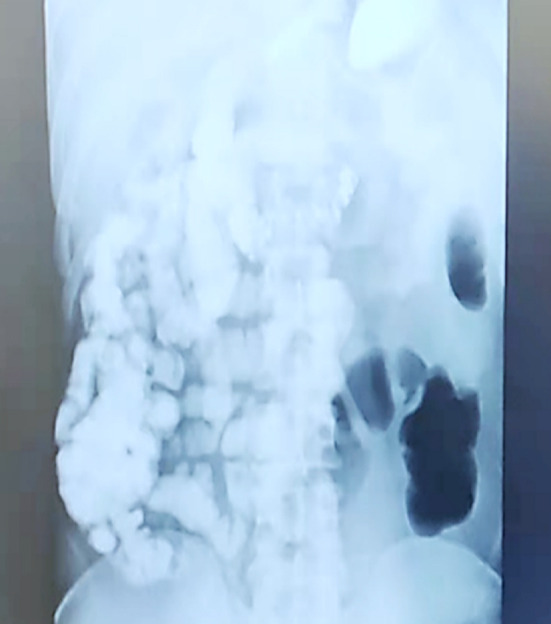
Full gastrointestinal contrast study (with iodized oil) shows gas accumulation in the left lower colon and partial small intestine.

The patient was advised to refrain from eating or drinking, and electrolyte imbalances were corrected. Fluid replacement, spasmolytics, inhibition of gastric acid secretion, pain relief, and symptomatic treatment were provided. Colonoscopy did not reveal any abnormalities. A full gastrointestinal contrast study (with iodized oil) showed gas accumulation in the left lower colon and partial small bowel (Fig.2). Abdominal CT with contrast enhancement indicated good perfusion of the mesenteric vasculature without apparent vascular abnormalities. A consultation with the mental health department considered the possibilities of Intellectual developmental delay and Depressive state.

**Fig.3:**
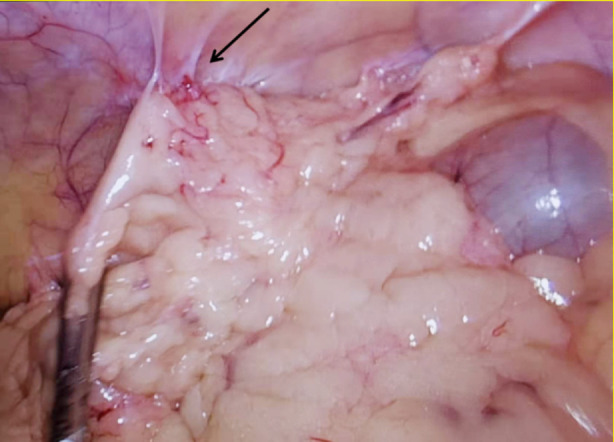
Adhesions between the greater omentum and the left lower abdominal wall, causing compression of the descending colon and resulting in obstruction.

On the 7^th^ day of hospitalization, the patient experienced recurrent abdominal pain following a meal. The pain was intermittent and localized to the left lower abdomen without radiation to other areas. He also had accompanying symptoms of nausea and non-projectile vomiting, with vomitus containing gastric contents. There was no fever, chills, diarrhea, passing of gas, or bowel movements. After thorough communication with the family, a diagnostic laparoscopy was performed. Intraoperatively, adhesions were observed between the greater omentum and the left lower abdominal wall (Fig.3). These adhesions were compressing the colon, leading to the obstruction. The proximal descending colon was dilated to approximately 7cm in diameter. After releasing the adhesions, the intestinal contents were able to pass into the distal descending colon, relieving the obstruction. The diagnosis of obstruction at this site was confirmed. The patient had an uneventful recovery after the surgery, was able to tolerate oral intake, and had normal bowel movements. During a 6-month follow-up, there were no further episodes of abdominal pain.

## DISCUSSION

Surgery is the most effective treatment for HSCR. In this case, the patient initially showed improvement with treatments, including fasting, fluid replacement, and nutritional support during the acute episode. However, the symptoms recurred after oral intake, and no significant structural abnormalities were found. Due to the recurrent abdominal pain, a diagnostic laparoscopy was performed. During the surgery, adhesions were identified between the greater omentum and the left lower abdominal wall, which were compressing the descending colon and causing obstruction. After releasing the adhesions, the obstruction was relieved as intestinal contents were able to pass into the distal colon. There have been reports of intestinal obstruction in adult DS patients due to malrotation of the midgut^5^, as well as cases of obstruction caused by ileocecal colonic torsion.^6^,^7^ In this particular patient, there was no history of previous surgery or trauma, and the cause of adhesions between the greater omentum and the abdominal wall remains unclear. Further research is needed to explore any potential correlation between DS and these adhesions. Hence, when evaluating intestinal obstruction in adult DS patients, consideration of multiple factors is necessary, and diagnostic laparoscopy may be required to determine the underlying cause.

Patients with DS commonly have congenital defects, including cardiac abnormalities (40%-50%) and gastrointestinal malformations (approximately 12%).^8^ They have a genetic predisposition to intestinal abnormalities, with up to 77% of DS children having associated gastrointestinal abnormalities, which can be either structural or functional in nature.^9^

DS is closely associated with gastrointestinal malformations. Common upper gastrointestinal abnormalities include duodenal atresia and annular pancreas, while common lower gastrointestinal abnormalities include Hirschsprung’s disease (HSCR) and anorectal malformation.^10^ Among cases of prenatal diagnosis of duodenal atresia, 46% are associated with DS.^11^ HSCR is present in 2%-15% of individuals with DS, compared to a general population incidence of 0.15%-0.17%.^12^ DS patients have higher levels of collagen VI in the surrounding tissues of the enteric ganglia compared to healthy controls (threefold increase). Elevated collagen VI levels can reduce the migration speed of enteric neural crest-derived cells involved in the formation of the enteric nervous system, leading to the absence of the enteric nervous system in the distal intestine and the occurrence of HSCR.^13^ In this case, the patient complained of abdominal distension and had tenderness on palpation in the left lower abdomen, which needed to be differentiated from HSCR. Approximately 50% of DS patients have gastrointestinal abnormalities, with chronic constipation being the most common, and HSCR often presents with a history of constipation. Barium enema and CT imaging clearly show the absence of ganglion cells in the narrowed segment of the intestine and dilated segments, which is not supported by the CT and gastrointestinal imaging in this patient.

### Insights from this case:

- Patients with intellectual disabilities may experience delayed or missed diagnosis and potentially fatal consequences due to difficulties in effective communication. There are reports of significant delays in diagnosing DS patients with paraumbilical hernias, and the possibility of paraumbilical hernias should be considered in DS patients with recurrent hospital admissions due to chest infections.^14^ There is also a reported case of a 35-year-old man with DS who died in a nursing facility. An autopsy revealed necrosis due to torsion in the appendix, ascending colon, and hepatic flexure, with a maximum diameter of the dilated intestinal segment reaching 15 cm.^15^
- Caregivers of patients with intellectual disabilities should have some medical knowledge, and healthcare professionals need to approach the diagnosis and treatment of patients with mental disorders with caution and thoroughness.
- In this particular case, the patient had the intellectual capacity of a 7-8 years old child, and the disease had been present for two years before diagnosis and treatment. The difficulties in communication and a lack of awareness among healthcare professionals resulted in a delay in initiating appropriate treatment. However, thanks to the dedicated care and proactive efforts of the patient’s family in seeking medical assistance, successful treatment was eventually achieved. Paying attention to the specific needs of these patient populations can significantly enhance their quality of life and have a positive impact on both the patients and their families. This approach holds significant social significance.

### Limitations:

This study is based on a case report, and limited persuasive conclusions. Further intervention trials are needed in the future to confirm these results.

## CONCLUSIONS

In summary, this case highlights a potential association between Down syndrome and congenital abdominal adhesions that can lead to intestinal obstruction. Due to the communication difficulties in patients with Down syndrome, there is a potential for misdiagnosis, emphasizing the importance of awareness. Laparoscopic examination not only aids in the diagnosis but also offers therapeutic benefits in such cases.
